# Construction and validation of educational material for the health promotion of individuals with HIV*

**DOI:** 10.1590/1518-8345.3748.3322

**Published:** 2020-09-07

**Authors:** Giselle Juliana de Jesus, Juliano de Souza Caliari, Layze Braz de Oliveira, Artur Acelino Francisco Luz Nunes Queiroz, Rosely Moralez de Figueiredo, Renata Karina Reis

**Affiliations:** 1 Universidade de São Paulo, Escola de Enfermagem de Ribeirão Preto, PAHO/WHO Collaborating Centre at the Nursing Research Development, Ribeirão Preto, SP, Brazil.; 2 Instituto Federal do Sul de Minas Gerais, Enfermagem, Passos, MG, Brazil.; 3 Universidade Federal de São Carlos, Departamento de Enfermagem, São Carlos, SP, Brazil.

**Keywords:** HIV, Teaching Materials, Validation Studies, Health Literacy, Self Care, Nursing, HIV, Materiais de Ensino, Estudos de Validação, Alfabetização em Saúde, Autocuidado, Enfermagem, VIH, Materiales de Ensenanza, Estudios de Validación, Alfabetización en Salud, Autocuidado, Enfermería

## Abstract

**Objective:**

to develop and validate an educational technology for individuals living with the human immunodeficiency virus.

**Method:**

a methodological study, for the elaboration of educational material. The educational needs, content selection, and illustrations were defined from interviews with the target population. Afterward, we carried the writing, the material layout elaboration, and assembly and, subsequently, it was validated by specialists. The content validation was established from the Level Content Validity Index higher than 0.8.

**Results:**

the educational material was prepared for adults living with the human immunodeficiency virus, with a focus on health promotion and quality of life, and was prepared in five volumes. The validation was made by 22 multi-professional judges selected according to the criteria established in the study. All items were evaluated as relevant by the judges and the average obtained with the index was 0.97.

**Conclusion:**

the booklet has been validated in terms of content, language, and appearance by experts in the field. We believe that through this technology it is possible to contribute to the health literacy and empowerment of individuals living with the human immunodeficiency virus, strengthening their autonomy.

## Abstract (pt)

**Objetivo:**

desenvolver e validar uma tecnologia educacional para pessoas vivendo com o vírus da imunodeficiência humana.

**Método:**

estudo metodológico, para a elaboração do material educativo. Definiram-se as necessidades educacionais, seleção dos conteúdos e ilustrações, a partir de entrevistas com a população-alvo. A seguir, realizou-se a redação, elaboração e montagem do layout do material e posterior validação por especialistas. A validação de conteúdo foi estabelecida a partir do Level Content Validity Index maior que 0,8.

**Resultados:**

o material educativo foi elaborado para adultos vivendo com o vírus da imunodeficiência humana com o enfoque na promoção da saúde e qualidade de vida e foram elaborados em cinco volumes. A validação foi realizada por 22 juízes multiprofissionais selecionados de acordo com os critérios estabelecidos no estudo. Todos os itens foram avaliados como pertinentes pelos juízes e a média obtida com o referido índice foi de 0,97.

**Conclusão:**

a cartilha elaborada foi validada quanto ao conteúdo, linguagem e aparência junto a especialistas na temática. Acredita-se que, por meio desta tecnologia, é possível contribuir com a alfabetização em saúde e o empoderamento das pessoas vivendo com o vírus da imunodeficiência humana, fortalecendo sua autonomia.

## Abstract (es)

**Objetivo:**

desarrollar y validar una tecnología educativa para personas que viven con el virus de la inmunodeficiencia humana.

**Método:**

estudio metodológico para la elaboración de material educativo. Se definieron necesidades educativas, selección de contenido e ilustraciones, a partir de entrevistas con la población objetivo. Luego, se llevó a cabo la redacción, elaboración y montaje del diseño del material y posterior validación por parte de especialistas. La validación de contenido se estableció a partir del Level Content Validity Index superior a 0,8.

**Resultados:**

el material educativo fue desarrollado para adultos que viven con el virus de la inmunodeficiencia humana con enfoque en la promoción de la salud y la calidad de vida y se desarrolló en cinco volúmenes. La validación fue realizada por 22 jueces multiprofesionales seleccionados de acuerdo con los criterios establecidos en el estudio. Todos los ítems fueron evaluados como pertinentes por los jueces y el promedio obtenido con el índice referido fue de 0,97.

**Conclusión:**

el folleto elaborado fue validado en términos de contenido, lenguaje y apariencia junto a especialistas en el tema. Se cree que, a través de esta tecnología, es posible contribuir a la alfabetización en salud y al empoderamiento de las personas que viven con el virus de la inmunodeficiencia humana, fortaleciendo su autonomía.

## Introduction

More than three decades after the discovery of the human immunodeficiency virus (HIV) infection, this pandemic remains a global public health problem. It is estimated that in 2017 there were 1.8 million new HIV infections worldwide, totaling 36.9 million people living with HIV, 1.8 million of them in Latin America and the Caribbean alone. Brazil is considered the largest country in Latin America in cases of new HIV infections^(^ 1 ^-^ 2 ^)^. 

There is a consensus on understanding HIV as a chronic and incurable infection. But with the advent of antiretroviral therapy (ART), individuals living with HIV (ILWHA) have improved their living conditions by reducing hospital admissions and opportunistic infections, positively impacting on the reduction of mortality and the reduction of AIDS progression rates among those infected with HIV^(^ 3 ^)^. 

From the perspective of assisting chronic patients, HIV care requires new skills on the part of health care professionals, as well as a redesign of health care systems that were initially designed for acute care. Studies point out the importance of comprehensive care to these patients, as well as their empowerment, strengthening their autonomy and responsibility regarding the treatment progress^(^ 4 ^-^ 5 ^)^.

Thus, studies show that health education is an effective means that can contribute to this debate and subsidize interventions for improving the ILWHA quality of life. Educational strategies are one of the adherence-to-treatment pillars, as it is for the prevention of the HIV spread, and proposes well-being subsidies^(^ 6 ^-^ 7 ^)^.

The use of educational material is characterized by being an emancipatory technology, especially by the possibility of allowing ILWHA to change their attitude and adhere to preventive practices since it brings information capable of acting on the empowerment of individuals, by enabling them to learn and activate their potential for self-care and favoring the process of communication and guidance among the health team, patients and relatives^(^ 8 ^)^.

In Brazil, although studies have described a variety of educational materials as an educational tool in various settings, populations, and purposes^(^ 8 ^-^ 12 ^)^, there is still a need to build and validate educational materials for adult ILWHA through applied and theoretically based planning, aiming to provide health information in a meaningful way to ensure the empowerment of ILWHA, strengthening their autonomy and responsibility for the progress of treatment, so that they can understand their health and make informed decisions in achieving a better quality of life.

Thus, the objective of this study was to develop and validate an educational technology for individuals living with the human immunodeficiency virus. 

## Method

This is a methodological study for the construction and validation of an educational booklet, developed in two stages from January 2015 to June 2017: the first was the planning that included the definition and identification of the educational needs of the target population, content selection, and illustrations. The second one consisted of the writing and layout designing of the educational material and validation by judges. 

In order to identify the educational needs of the target population, users of two specialized HIV/AIDS services in a municipality in the upstate São Paulo were approached.

In the first stage of the study, 26 HIV/AIDS-positive individuals who were in follow-up in the selected services participated in the study. Individuals aware of their HIV seropositivity, 18 years of age or older were included, and who were in clinical and ambulatory follow-up at the chosen services. 

The data collection took place through a semi-structured interview with sociodemographic and clinical characterization variables to survey the profile of the participants and a script with guiding questions, with the purpose of identifying doubts and also positive and negative aspects related to living with HIV/AIDS.

The sample was consecutive and the saturation of the data was used as a criterion for the finalization of the collection^(^ 13 ^)^. 

To process the qualitative data, we used the lexical type analysis technique, with the help of the *software Interface de R pour les Analyses Multidimensionnelles de Textes et de Questionnaires* (IRaMuTeQ), the details of this analysis have already been described above^(^ 14 ^)^. 

In the second stages, we performed a review of the scientific literature, as well as of manuals and guidelines from the Ministry of Health and other institutions such as the Brazilian AIDS Association (Associação Brasileira de AIDS, ABIA) and the Joint United Nations Programme on HIV/AIDS (UNAIDS). The search for this material focused on the selected topics about the educational needs of the participants and also focused on the promotion of health and quality of life. 

The construction of this material has as theoretical reference the stages of learning from the Social Cognitive Theory by Bandura (1997)^(^ 14 ^)^ and the Health Literacy, and followed the methodological steps by Doak, Doak, and Root (1996)^(^ 15 ^)^. In order to prepare the educational material, we adopted the recommendation on the use of common words, the inclusion of examples to explain complex guidelines and interaction with the target population, since these steps make it possible to write an educational material that is understandable to this population^(^ 15 ^)^.

The content was then diagrammed by one communication and graphic *design* professional with experience in preparing educational material for patients and images by photographs. 

The photographs were taken by two professional photographers and sought to capture images that were attractive and appropriate to the target audience and that referred to situations related to the topics, in order to motivate reading. Besides the care with the language, the textual elaboration, the disposition of the images and the organization of the items of each page followed the steps that drive the Social Cognitive Learning, outlined by Bandura in 1997^(^ 14 ^)^.

For the validation of the educational material, a committee of experts was constituted, composed of researchers and teachers on the areas of HIV/AIDS, educational technologies and/or instrument validation In order to establish parameters for the election of the participants, the following criteria were adopted for the selection of specialists: to have clinical experience, to do research and publish on the issue^(^ 15 ^)^, to be an expert in the conceptual structure involved and have knowledge of building/evaluating educational material proven in the Lattes curriculum.

There is no consensus in the literature on the number of judges needed for a validation study. In this study, the sample calculation for determining the number of expert judges took place according to the formula for a proportion-based sample calculation^(^ 16 ^)^ n= Zα².P(1-P)\e². In the formula: “Zα²” is the confidence level adopted; “P” the expected proportion of experts who agree with each item evaluated; and “e” refers to the acceptable proportional difference from what is expected. The confidence level of 95%, the coefficient Zα of 1.96, the proportion of 85% of specialists and a difference (error) of 15% were adopted^(^ 16 ^)^. Thus, according to the above, the final estimated sample consisted of 22 specialists. However, in order to reach the estimated sample, 44 invitations had to be made, of which 50% were able to attend to the invitation.

The judges were invited via e-mail; and after confirmation of interest in participating in the study, they received in their respective e-mails the *link* to the questionnaire built and hosted in the Google Forms and a pdf copy of the booklet. 

The questionnaire sent to the judges for evaluation of content and appearance consisted of 30 Likert type items distributed in seven evaluation aspects: two of content (scientific accuracy and content) and five of appearance (language, illustrations, layout, motivation, and culture), all based on the Suitability Assessment of Materials^(^ 17 ^)^. 

For each topic in the booklet, the judges assessed the adequacy and presentation of the information considering the readers’ perspective with respect to reading motivation and cultural aspects. Regarding content, they assessed whether it was addressed based on current knowledge, whether the guidelines presented were necessary, and whether the technical terms were adequately defined. With regard to language, the judges assessed the convenience and ease of understanding and whether the most important concepts were addressed with clear and objective vocabulary. As for the illustrations, they evaluated the adequacy of the visual composition, their attractiveness and organization, as well as the quantity and adequacy of the illustrations.

The questionnaire data about the validation was tabulated in the Microsoft Excel program, and the data analysis took place based the Content Validation Index (CVI). The levels of agreement and relevance of each item ranged from 1 to 4 (1-totally disagrees; 2-partially agrees; 3-agrees; and 4-totally agrees). For each item in the questionnaire, a numeric value has been added so that for the options “totally agrees” and “agrees”, the value +1 has been added because they are positive assessments; for the option “partially agrees”, the value 0 (zero) has been added because it is a partial option; and for the options “totally disagrees”, the value -1 has been assigned because it is a negative assessment option. From these values, the CVI was calculated. 

The level Content Validity Index (I-CVI) was used to evaluate the level of agreement among the judges for each item. I-CVI was computed by the number of judges who evaluated the item as relevant and very relevant. The Scale-Level Content Validity Index, Average Calculation Method (S-CVI/AVE) was calculated using the proportion of the items in the scale assessed as relevant and very relevant by each judge. The item with an index equal to or greater than 0.80 was considered validated. To analyze the proportion of agreement on the adequacy and relevance of the booklet, it was statistically equal to or greater than 0.8, we did the binomial test, with a significance level of 5%^(^ 18 ^)^.

The study was approved by the Research Ethics Committee of the Ribeirão Preto Nursing School, University of São Paulo (CAEE 55081716.1.0000.5393; no. of the opinion 1.635.190) and complied with the ethical principles in the Resolution 466/12. 

## Results

We built an instructional printed educational material. The first version of the booklet was a material containing 77 pages of content, organized in a questions-and-answers format without text diagramming and insertion of photographs. After the diagramming, the material had 212 pages divided into 05 volumes, size approximately half an A4 sheet - 27.2 cm. 

**Figure 1:**
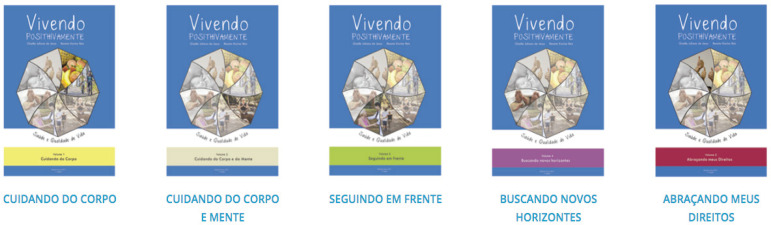
Booklet volumes. Ribeirão Preto, SP, Brazil, 2017

The final version of the booklet was printed in 4x4 color printing, the cover in a 170 g glossy couche paper, and the kernel in 115 g glossless *couché* paper. The volumes were affixed with staples, in half A4 sheet size, 27.2 cm. Composed of cover, cataloging and technical sheet, cover sheet, summary, presentation, introduction to the key theme with dedication, preface, acknowledgments, consulted references, notes, and back cover, as exemplified in Figure 1. 

From the results of the first stage with the help of the IRaMuTeQ software, based on the Descending Hierarchical Classification (CHD), the most relevant words in the testimonials were analyzed and their relationship with the objective of the booklet, resulting in five content classes, which helped to guide the booklet’s division and organization in their respective volumes. 

In the sample of 26 ILWHA, 13 (50%) were men and 13 (50%) women with ages ranging from 28 to 73 and a mean of 50 years, and 22 (84.6%) reported being heterosexual and 15 (57.7%) had no sexual partnership at the time of the interview. Of the total, nine (34.6%) mentioned being single. We identified that the majority of the participants, 17 (65.4%), had up to eight years of study and individual income of up to two minimum wages. 

The educational needs on health and quality of life have contemplated various aspects of living with HIV/AIDS, pointing out the complexity of living with a chronic condition that triggers a diversity of feelings and behaviors and imposes changes in the daily life of ILWHA, but also in their family and social and personal relationships. 

The target population was included in several stages in the development of the educational material, based on the relevance of developing educational material in line with their needs and perceptions of ILWHA, respecting the cultural characteristics and differences of this population.

Throughout the process of building the booklet, there was attention towards the suitability of language, by identifying the technical terms and transforming them into a popular and simple language, aiming to facilitate reading and understanding by the ILWHA. 

To ensure this, several readings of the content were carried out to identify technical terms and replace them with simpler explanations, common words or examples, in addition to including a Portuguese language teacher among the judges. Another aspect considered fundamental was the inclusion of photographs to motivate reading and make the content easily understandable. 

After the first contact with the participants and identification, by means of the interview, of the doubts on living with HIV, we carried the second stage, which was characterized by the search for available scientific articles, guidelines recommended for the adult population living with HIV/AIDS, dissertations available on the thesis portal of the Coordination for the Improvement of Higher Education Personnel and textbooks in order to provide input for the development of the booklet topics and their content.

We entered the following keywords in the search: “nutrition” (“nutrição”), “physical exercise” (“exercício físico”), “treatment” (“tratamento”), “confrontation” (“enfrentamento”), “revelation” (“revelação”), “secrecy of seropositivity” (“sigilo da soropositividade”), “right to have children” (“direito de ter filhos”), “sexual practices” (“práticas sexuais”), and descriptors from the Descritores em Ciência da Saúde/Medical Subject Headings (DeCS/MeSH): “health promotion” (“promoção da saúde”), “quality of life” (“qualidade de vida”), “exercise” (“exercício”), “therapeutics” (“terapêutica”), “mental health” (“saúde mental”), “adaptation, psychological” (“adaptação psicológica”), “leisure activities” (“atividades de lazer”), “self concept” (“autoimagem”), “sleep” (“sono”), “rest” (“descanso”), “disclosure” (“revelação”), “confidentiality” (“confidencialidade”), “HIV seropositivity”, (“soropositividade para HIV”), “sexuality” (“sexualidade”), “sexual partners” (“parceiros sexuais”), “risk” (“risco”), “transmission” (“transmissão”), “prevention” (“prevenção de doenças”), “counseling” (“aconselhamento”), “serologic tests” (“testes sorológicos”), “post-exposure prophylaxis” (“profilaxia pós-exposição”), “reproduct”, “right” (“direitos sexuais e reprodutivos”), “right” and “health” (“direito à saúde”), associated through the Boolean operator AND. Works published from 2011 to 2016 has been included.

After the diagramming and insertion of the photographs, we decided to organize all the content in a more didactic way and to divide the educational booklet into five volumes entitled: Volume 01 - Caring for the Body, Volume 02 - Caring for the Body and Mind, Volume 03 - Moving Forward, Volume 04 - Seeking New Horizons, and Volume 05 - Embracing My Rights. In the volumes, several different topics were addressed in total (food and physical exercise, mental health promotion, treatment and coping with HIV diagnosis, sexuality, sexual health and prevention, and rights of individuals living with HIV/AIDS, and they can be accessed online, in PDF, and http: http://gruposdepesquisa.eerp.usp.br/sites/cartilha/ (Figura 1).

The educational material content validation was carried out by a committee of 22 specialists, composed of a multi-professional team formed by a physician, nurse, nutritionist, psychologist, pharmacist, social worker, and physical educator. Regarding the occupation, 14 (63.6%) judges were professors and researchers; 09 (40.9%) practiced exclusively their activities in higher education institutions, 07 (31.8%) had experience in the welfare area. The options agrees or totally agrees were marked by 100% of the judges on 17 items, by 95% of the judges on 11 items and by 95% of the judges on 2 items. Thus, the I-CVI of each item was calculated, with a mean of 0.97 (Table 1).

**Table 1:** Judges' agreement on the items in the booklet. Ribeirão Preto, SP, Brazil, 2017

	D*	PA^†^	A^‡^	TA^§^	I-CVI^||^
**1. Scientific accuracy**					
CVI^¶^ - Total	0	4	20	42	**0.93**
**2. Content**					
CVI^¶^ - Total	0	3	41	132	**0.98**
**3. Language**					
CVI^¶^ - Total	0	5	59	134	**0.97**
**4. Illustrations**					
CVI^¶^ - Total	0	3	17	46	**0.95**
**5. *Layout***					
CVI^¶^ - Total	0	0	5	39	**1**
**Learning Stimulation/Motivation**					
CVI^¶^ - Total	0	0	18	70	**1**
**7. Culture**					
CVI^¶^ - Total	0	0	2	20	**1**
**Mean**					**0.97**

* D = totally disagrees;

† PA= partially agrees;

‡ C = agrees;

§ TA = totally agrees;

|| I-CVI = level Content Validity Index;

¶ CVI =Content Validity Index

As for the adequacy of the educational material, the I-CVI mean of agreement between the judges was 0.95: 01 S-CVI for the scientific accuracy domain and 0.98 S-CVI for the content domain. The relevance proportion (S-CVI/AVE) of the 30 items of the instrument was 100% among 15 judges and for only one the S-CVI/AVE was 0.73. S-CVI of 0.97 was obtained and the I-CVI of each item evaluated separately was higher than 0.80 (Table 1). 

In the validation of the booklet, there was 100% agreement of the judges on the content “to be understood” and 95% on the content “to be relevant” and meet possible needs of the target audience, which makes the educational material applicable. The judges demonstrated a positive evaluation of the booklet and indicated the material as an excellent resource for ILWHA to consult as a source of evidence-based information appropriate for the care of their health and quality of life. 

The proportion of relevance (S-CVI/AVE) of the 30 items of the instrument was 100% among 15 judges, and from only one the S-CVI/AVE got 0.73. The S-CVI got 0.97 and the I-CVI of each item evaluated separately was higher than 0.80.

The S-CVI/AVE was calculated for each judge and from their mean, the S-CVI was calculated as shown in Table 2.

**Table 2:** Judges' agreement on the booklet's proportion of relevance. Ribeirão Preto, SP, Brazil, 2017

Judge	D*	PA^†^	A^‡^	TA^§^	S-CVI/AVE^||^
**1**	0	1	9	20	0.96
**2**	0	0	1	29	1
**3**	0	0	3	27	1
**4**	0	0	4	26	1
**5**	0	0	11	19	1
**6**	0	0	14	16	1
**7**	0	1	3	26	0.96
**8**	0	1	3	26	0.96
**9**	0	0	3	27	1
**10**	0	0	0	30	1
**11**	0	0	5	25	1
**12**	0	0	23	7	1
**13**	0	2	20	8	0.93
**14**	0	0	0	30	1
**15**	0	1	2	27	0.96
**16**	0	8	16	6	0.73
**17**	0	0	3	27	1
**18**	0	0	4	26	1
**19**	0	0	2	28	1
**20**	0	0	10	20	1
**21**	0	0	0	30	1
**22**	0	1	14	15	0.96
**S-CVI** ^¶^					**0.97**

* D = totally disagrees;

† PA= partially agrees;

‡ A = agrees;

§ TA = totally agrees;

|| S-CVI/AVE = relevance proportion;

¶ I-CVI = level Validity Content Index

The agreement among the judges on the adequacy and relevance of the booklet was significant for all except for the judges 13 and 16 who most frequently indicated partially agrees (Table 3).

**Table 3:** Assessment of the agreement among the judges on the booklet's adequacy and relevance. Ribeirão Preto, SP, Brazil, 2017

Judges	Estimate	p-value*	95% CI^†^	95% CI^†^
1	0.96	0.04	0.85	1.00
2	1.00	0.01	0.90	1.00
3	1.00	0.01	0.90	1.00
4	1.00	0.01	0.90	1.00
5	1.00	0.01	0.90	1.00
6	1.00	0.01	0.90	1.00
7	0.96	0.04	0.85	1.00
8	0.96	0.04	0.85	1.00
9	1.00	0.01	0.90	1.00
10	1.00	0.01	0.90	1.00
11	1.00	0.01	0.90	1.00
12	1.00	0.01	0.90	1.00
13	0.93	0.15	0.80	1.00
14	1.00	0.01	0.90	1.00
15	0.96	0.04	0.85	1.00
16	0.73	0.97	0.57	1.00
17	1.00	0.01	0.90	1.00
18	1.00	0.01	0.90	1.00
19	1.00	0.01	0.90	1.00
20	1.00	0.01	0.90	1.00
21	1.00	0.01	0.90	1.00
22	0.96	0.04	0.85	1.00

* Binomial Test;

† 95% confidence interval for the parameters

We point to the fact that although the result of the CVI-Total of all the domains is above 0.8, we chose to follow all the suggestions of the judges for the booklet in the presentation of the final version.

## Discussion

The construction of the educational material was based on the profile and educational needs of the target population. Thus, considering these characteristics, the educational material for guidance to the ILWHA has been directed at reaching adults of both sexes, with different levels of literacy, including from individuals with few years of study (primary education) to the ones with a high level of schooling (higher education). 

It is very important to profile the target population before developing a health education material, for the materials usually have a mismatch between the instructions and the individuals to whom they are directed, although they are widely used in various aspects of health care^(^ 18 ^)^.

The contents that subsidized the construction of the educational booklet included several themes involving aspects related to physical and mental health, sexual practices, family planning, confronting the stigma, discrimination, and the rights of individuals living with HIV/AIDS. 

In the volume 1, topics on nutrition, benefits of healthy eating, and physical activity for improving immunity were presented. The choice of topics related to body care is a concern already described in other studies by ILWHA, with healthy eating and physical activity being pointed out as part of healthy self-care, important elements for the QL of participants^(^ 19 ^)^.

The maintenance of a balanced and healthy diet, as well as physical activity, are considered to be care measures that contribute to health promotion and are fundamental to keep up physical and emotional health. Such health practices provide improved QL for the individuals, lowering mortality rates and increasing adherence to antiretroviral treatments, which are directly linked to improving the immune system^(^ 20 ^)^.

In volume 02, we approached the care of body and mind. The physical impacts of the TARV have long been the main concern on the impact of QL on ILWHA, however as new generations of drugs have been implanted in the health system, we could perceive a decrease in the prevalence of the most severe symptoms^(^ 21 ^-^ 22 ^)^. Nevertheless, there has been a dramatic growth in symptoms of mental malaise among ILWHA over the years, especially in developing countries^(^ 23 ^)^. 

These mental health problems can also arise as a side effect of antiretroviral treatment or the stigma, stress, and socioeconomic situations associated with the infection and treatment process. At the same time, depression and substance use disorders, which commonly occur together, can increase the chance of behaviors that promote HIV transmission, such as risky sexual activity and injecting drug use. These associations are usually found in cross-sectional studies, so that understanding which aspects really determine this relationship is even more complex^(^ 18 ^)^.

Volume 03 focused on moving forward. It is well known that the impact of the diagnosis still sharpens feelings of doubt, uncertainty, insecurity and lack of support mainly due to the stigma imbedded in cultural roots of HIV history, in this perspective, the knowledge of ILWHA through educational technologies strengthens their empowerment and helps in decision making, providing a foundation for a biopsychosocial well-being^(^ 8 ^)^.

The intimidation and embarrassment of living with this chronic condition still limit discussions within the family, social, and even health services environments, and the disposition of these new interventions can allow the user to understand the new perspectives of living with HIV and to clear up doubts on the subject.

In volume 04, the search for new horizons was addressed - aspects of sexuality, affective-sexual life and sexual practices and risk of HIV transmission, preventive practices that included the use of the male and female condom, counseling couples, HIV testing between sexual partnerships and use of Post-exposure Prophylaxis (PEP) to HIV. 

The way sexual minorities are differently affected by HIV and its impact on the quality of life^(^ 6 ^)^, has motivated this study to take into account the diversity of affective-sexual partnerships, both in the content and representations (photos and illustrations).

Still, in volume 05, rights were addressed: the desire to have children, family planning, and the fundamental rights for ILWHA. And although HIV brings fears and apparent limitations to ILWHA^(^ 24 ^)^, the desire for plans related to maintaining or building affective-sexual and family relationships, as identified in this study, is legitimate and can be found in others studies with different populations of ILWHA, such as men who have sex with men(24), pregnant woman^(^ 25 ^)^ and serodifferent couples^(^ 26 ^)^.

The reproductive rights of ILWHA are the same for those not infected by the virus. However, we perceive that such rights can be oppressed by a lack of information related to transmission, fear, and stigma in the face of perceived guilt for the condition^(^ 27 ^)^.

Thus, we highlight the importance of educational materials, like the one developed in this study, which comes to collaborate with the increase of information for ILWHA, empowering them in the search for the accomplishment of their rights^(^ 28 ^)^. 

In the validation of the booklet, there was 100% agreement of the judges that the content will be understood and 95% that the content will be relevant and meets the possible needs of the target audience, which makes the educational material applicable. This agreement of the judges on the applicability of the material is observed in other studies of educational booklet validation^(^ 8 ^,^ 29 ^)^. 

The criterion of understanding, relevance, and applicability of educational material is of paramount importance since it is not enough for educational material to have valid and understandable content. If it is not applicable, it is, therefore, necessary to critically rethink all the material.

The judges showed a positive evaluation of the booklet and indicated the material as an excellent resource for ILWHA to consult within and outside the health environment. Moreover, the collection was considered a complement for the practical guidelines by health professionals on the subject, mainly because of the style with which the content was added and, subsequently, organized: once exposed in a conversational way, organized in a question-and-answer format, the target audience, when reading the material, can then feel more motivated to follow the proposed guidelines. 

Translating technical and scientific language into a language accessible to the population, particularly to those with lower health literacy, is a challenge. The development of the booklet as educational material from the educational needs and with the participation of the target population was a fundamental strategy in this study. We thus hope that this technology will facilitate communication and access to information amongst ILWHA and the healthcare team. 

As a limitation, we point out the fact that in the first stage, the study included only ILWHA who were linked to health services and with participants from only one region of the country. In this way, there may be differences in educational needs in other cultural and social realities and contexts. Even so, the complementation of this stage with the literature sought to reduce this limitation. 

## Conclusion

The booklet was prepared out of the educational needs and with the participation of the target population and was validated in terms of content, language, and appearance by specialists in the field. We believe that through this technology it is possible to provide relevant information that can contribute to the health literacy and empowerment of ILWHA; strengthening their autonomy and responsibility in the face of scientific advances, and helping in the understanding of their health condition and decision making, aiming at achieving a better quality of life. Also, the use of validated material can enhance the educational practice of nurses and the multidisciplinary team. 

We also believe that the use of these technologies, especially illustrated with photos, helps to reduce the stigma attached to ILWHA and helps to increase information, allowing the user to understand the new perspectives of living with HIV and answer questions about the subject.

In the validation of the booklet, we identified the judges’ agreement on the understanding of the contents and the relevance in meeting the target audience, which makes the educational material applicable and an important intervention tool for health professionals and of access to the target population.

Finally, in order to analyze the contribution that the educational booklet can provide in the teaching-learning process of the target audience, mainly as an educational intervention in the context of comprehensive care for ILHIV, future studies will be carried out with a view to validating the appearance of the material and assessing the readability index by the ILWHA and its application in clinical practice. 

We finally highlight that the booklet was made available in the printed version for public health institutions and also for the target audience in its online version, as it can reach a higher number of ILWHA in the whole country.
